# Genome-wide identification of new Wnt/β-catenin target genes in the human genome using CART method

**DOI:** 10.1186/1471-2164-11-348

**Published:** 2010-06-01

**Authors:** Christian Hödar, Rodrigo Assar, Marcela Colombres, Andrés Aravena, Leonardo Pavez, Mauricio González, Servet Martínez, Nibaldo C Inestrosa, Alejandro Maass

**Affiliations:** 1 Laboratorio de Bioinformática y Expresión Génica, INTA, Universidad de Chile, Santiago, Chile; 2 Laboratorio de Bioinformática y Matemática del Genoma, Centro de Modelamiento Matemático (UMI 2807, CNRS), Facultad de Ciencias Físicas y Matemáticas, Universidad de Chile, Santiago, 8370459, Chile; 3 Departamento de Ingeniería Matemática, Facultad de Ciencias Físicas y Matemáticas, Universidad de Chile, Santiago, 8370459, Chile; 4 Centro de Regulación Celular y Patología "Joaquín V. Luco" (CRCP), MIFAB, Facultad de Ciencias Biológicas, Departamento de Biología Celular y Molecular, Pontificia Universidad Católica de Chile, Santiago, 8330025, Chile

## Abstract

**Background:**

The importance of *in silico *predictions for understanding cellular processes is now widely accepted, and a variety of algorithms useful for studying different biological features have been designed. In particular, the prediction of *cis *regulatory modules in non-coding human genome regions represents a major challenge for understanding gene regulation in several diseases. Recently, studies of the Wnt signaling pathway revealed a connection with neurodegenerative diseases such as Alzheimer's. In this article, we construct a classification tool that uses the transcription factor binding site motifs composition of some gene promoters to identify new Wnt/β-catenin pathway target genes potentially involved in brain diseases.

**Results:**

In this study, we propose 89 new Wnt/β-catenin pathway target genes predicted *in silico *by using a method based on multiple Classification and Regression Tree (CART) analysis. We used as decision variables the presence of transcription factor binding site motifs in the upstream region of each gene. This prediction was validated by RT-qPCR in a sample of 9 genes. As expected, LEF1, a member of the T-cell factor/lymphoid enhancer-binding factor family (TCF/LEF1), was relevant for the classification algorithm and, remarkably, other factors related directly or indirectly to the inflammatory response and amyloidogenic processes also appeared to be relevant for the classification. Among the 89 new Wnt/β-catenin pathway targets, we found a group expressed in brain tissue that could be involved in diverse responses to neurodegenerative diseases, like Alzheimer's disease (AD). These genes represent new candidates to protect cells against amyloid β toxicity, in agreement with the proposed neuroprotective role of the Wnt signaling pathway.

**Conclusions:**

Our multiple CART strategy proved to be an effective tool to identify new Wnt/β-catenin pathway targets based on the study of their regulatory regions in the human genome. In particular, several of these genes represent a new group of transcriptional dependent targets of the canonical Wnt pathway. The functions of these genes indicate that they are involved in pathophysiology related to Alzheimer's disease or other brain disorders.

## Background

Gene expression is the mechanism through cells organize which genes can be up-regulated or repressed in response to both, changes in their environment [1] or internal programs [2]. Primary control of gene expression occurs through transcriptional regulation of mRNA levels, where one or several transcription factor (TF) proteins recognize and bind specific motifs in the DNA, calling TF binding sites to modulate the activity of the basal transcriptional machinery [3]. In eukaryotes, the combination of TF binding sites clustered together near the transcription start sites in the promoter of genes are known as *cis *regulatory modules (CRMs), and changes in the combination of TFs bound to their respective binding sites contribute to up or down-regulate gene expression levels [4]. The computational identification of functional TF binding sites or CRMs is challenging. In general, methods that scan genome sequences to identify matches with a consensus binding site or a position weight matrix [5] produce high false positive rates owing to the low specificity of most of the profiles and the vast stretches of genomic sequences that have to be scanned. At the same time, prior knowledge has to be provided by the users to produce an unbiased genome survey of CRMs. New bioinformatics tools have been developed to avoid these problems in genome-wide predictions of mammalian *cis *regulatory regions. A novel method that involves binding affinity for TFs has been used to predict enhancers at the genomic scale [6]. Another strategy for *cis *regulatory modules prediction has been used in the analysis of human and mouse genomes based on finding phylogenetically conserved binding sites for different TFs [7,8]. The increasing amount of information available from microarray experiments [9,10] and TF binding sites [11,12] requires the development of new tools to improve our understanding of gene expression and transcriptional regulation. Based on the principle that co-expressed patterns emerge as the result of the combined action of TFs, several *in silico *methods have been used to elucidate the relationship between conserved motifs upstream of genes that are believed to be co-regulated [13,14]. These studies revealed that transcriptional regulation in eukaryotes works under a cooperative principle involving the binding sites for one or more TFs that are closely spaced in regulatory regions [15,16] and for which the composition is non-randomly distributed in various gene promoters [17]. The information arising from this kind of data allows to better understand how gene expression changes in response to environmental or internal programs, thereby connecting TF binding sites information with signaling pathways such as the Wnt pathway involved in many cellular processes.

The Wnt pathway is implicated in numerous aspects of development [18], cell differentiation [19-21], and several diseases [22,23]; notably, it was recently discovered a relation with cancer and neurodegenerative diseases like AD [24-26]. In the well-known Wnt pathway -- the highly conserved canonical Wnt/β-catenin signaling pathway [27] -- the secreted glycoprotein Wnt interacts with Frizzled, a seven-transmembrane receptor that transduces its signal through the activation of Dishevelled, which in turn inactivates GSK-3β kinase that resides in a protein complex assembled by the scaffolding protein Axin and *adenomatous polyposis coli *gene (*APC*) product, a tumor suppressor. As a consequence of the GSK-3β inactivation, hypophosphorylated levels of cytosolic β-catenin increase, allowing it to bind to components of the high mobility group family of transcription factors T-cell factor/lymphoid enhancer factor (TCF/LEF), and this complex is then translocated to the nucleus where it activates gene expression [28]. In the absence of the Wnt ligand, Axin is stabilized and the interaction between APC and β-catenin is facilitated. In this complex, β-catenin is phosphorylated by GSK-3β and destined for ubiquitin-proteasome-mediated degradation; as a result, the expression of Wnt signaling pathway target genes is repressed [29].

Several methods have been used to find new Wnt signaling pathway target genes based on the interaction between β-catenin and the evolutionarily conserved TCF/LEF, the most well known family of DNA binding factors involved in gene regulation through Wnt signaling: (1) reporter constructs based on TCF/LEF binding sites [30], (2) serial analysis of chromatin occupancy (SACO) [31] and (3) combined microarrays and chromatin immunoprecipitation (ChIP) [32]. All of these methods have disadvantages: reporter constructs shows discrepancies and may not reveal the complexity of gene regulation [33], and whole-genome SACO and ChIP strongly depend on high quality antibodies and represent just a particular point in the interaction between TFs and regulatory regions. In particular, these methods have been used with colon cancer cell lines, a more complex background to study TCF/LEF-dependent gene regulation, and predicted motifs for NF1, HNF4 or AP-1, among others, were discovered flanking TCF4 binding sites [32]. The method described by Hallikas et al. [6] was also used to identify targets for TCF4, a well-characterized member of the TCF/LEF family of TFs [34] downstream of the Wnt/β-catenin pathway, and suggested that Hedgehog signaling is involved in the Wnt pathway via GLI transcription factor motifs found close to the TCF4 binding sites.

Following the hypothesis that transcription factors work cooperatively to define gene expression, in this work we propose a multiple Classification and Regression Tree (CART) approach to identify new Wnt/β-catenin pathway target genes within the human genome, based on the presence of transcription factor binding site motifs in their regulatory regions. The CART method has already been used to classify acetylated or methylated promoters based on the binding site composition of a set of differentially expressed genes [35] or to identify relationships between gene expression levels and regulatory motifs in microarray experiments [36]. More directly, classification trees have been used to identify structural relationships between transcription factor binding sites and gene expression levels in order to discover regulatory motifs [36]. A similar approach was used to identify *cis *regulatory modules in differentially expressed genes of *D. melanogaster *germline [37].

In this work we used the predicted TF binding site motifs for a group of 15,476 genes in the human genome where 66 of them were known to be regulated by the Wnt/β-catenin pathway [38] to build a decision rule classifying genes as Wnt/β-catenin pathway target candidates based on the occurrences of these TF binding site motifs in their upstream regions.

## Results

### Generation of candidate genes to be Wnt/β-catenin pathway targets

**Figure 1:**
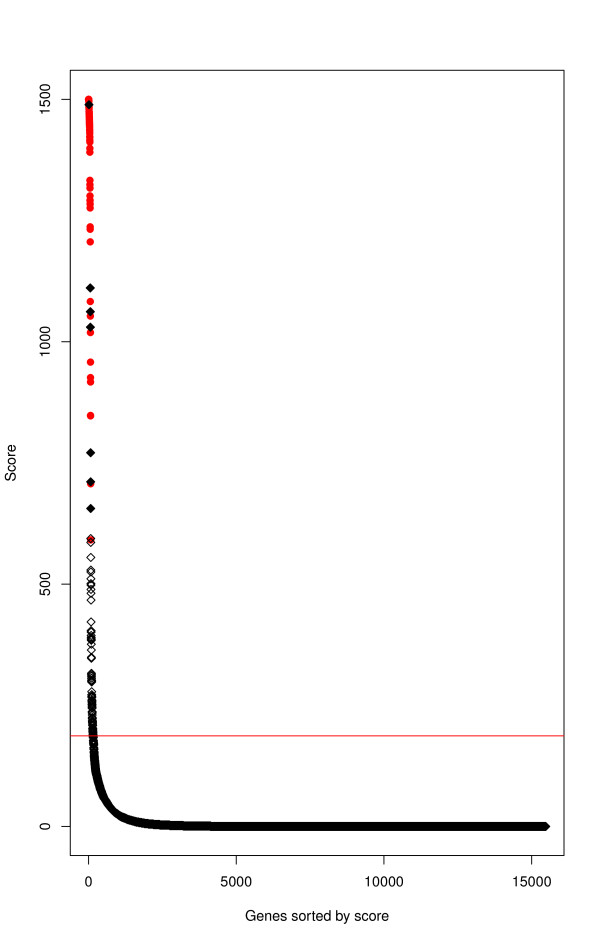
**Number of CART trees that declare a gene to be a Wnt/β-catenin pathway target**. Red points correspond to genes already known to be targets of the Wnt/β-catenin pathway, and black points represent genes not previously identified as Wnt/β-catenin pathway targets. The vertical axis denotes the "score" of each individual gene. Genes are ordered decreasingly with the score. The horizontal line represents the threshold value C associated to the highest percentile.

We trained independently a series of 1,500 CART trees using the information of the presence of transcription factor binding site motifs in the upstream region of 15,476 genes of the human genome where 66 of them are known to be Wnt/β-catenin pathway targets. We evaluated the 15,476 genes in the 1,500 CART trees and we ranked them using a "score" calculated as the number of times each one was classified as a Wnt/β-catenin pathway target among the 1,500 trees (Figure 1 and Additional File 1). Finally, a "threshold" value for the scores was proposed defining a "decision rule" to classify whether a gene is a Wnt/β-catenin pathway target. To define the score threshold we considered the highest percentile. Besides the 66 known target genes, this threshold produced 89 new candidates (Additional File 1). All previously known Wnt/β-catenin pathway target genes were correctly classified.

### Interpretation of the relevant transcription factors in the CART method

To provide a biological interpretation of the results generated by the method we studied the importance of variables in the classification, i.e. the usage of TF binding site motifs in the upstream region of the gene, and therefore the associated TFs. We used a primary index, giving more importance to variables appearing near the root in most of the 1,500 CART trees and a secondary index that just take the score or number of times that variables are used in the 1,500 trees. In Additional File 2 we provide a complete list of TF binding site motifs with the corresponding indexes and in Table 1 we provide the TFs associated to some of the variables considered to be the most relevant according to both indexes and a biological criterion. Finally both criteria revealed essentially similar results.

**Table 1:** A sample of relevant transcription factors

Entrez ID	Symbol	Name	I_1_	Score
2908	NR3C1 (GR)	*nuclear receptor subfamily 3, group C, member 1 (glucocorticoid receptor)*	822.6	1500
5077	PAX3	*paired box 3*	1389.7	1489
6932	TCF -1	*trascription factor 7*	3.3	1485
51176	LEF1	*lymphoid enhancer-binding factor 1*	68.5	1500
3172	HNF4a	*hepatocyte nuclear factor 4, alpha*	90.2	1497
4150	MAZ	*MYC-associated zinc finger protein (purine-binding transcription factor)*	6.3	1316
4520	MTF1	*metal-regulatory transcription factor 1*	27.2	1476

As expected, within the most relevant TFs used in the decision tree we found LEF1 and TCF-1. The complex formed between these regulator and β-catenin in upstream regions is necessary to regulate gene expression of canonical Wnt signaling pathway targets. Also, PAX3 transcription factor has been detected in vitro as part of a complex formed by LEF1 and repressor Grg4 in melanocyte stem cells [39]. The proposed model indicates a turnover between PAX3 and β-catenin for activation of dopachrome tautomerase gene (*Dct*) and the activation of a melanogenic cascade that leads to terminal differentiation of hair follicles. The presence of HNF4α transcriptional regulator as relevant for the predictor is also interesting. The study conducted by Hatzis et al. [32] revealed that binding sites motifs for this TF are present surrounding the specifically enriched TCF4-binding region identified by ChIP. In particular, Benahmed et al. [40] reported the cooperation between HNF4α, β-catenin and TCF-4 to regulate the expression pattern of the homeobox Cdx2 in mouse gut development. Recently it has been suggested that HNF4α could mediate gene expression of several drug transporter proteins in human and rat choroid-plexus [41]. Also recently, it has been demonstrated that transcriptional regulator GR (or NR3C1) is involved in down-regulation of cyclin D1 by targeting the TCF/β-catenin complex [42]; furthermore, it has been reported GR and β-catenin as part of the same immunocomplex in regulatory regions for cyclin D1 in human osteoblastic cells [43]. Regulatory sites for MAZ (also known as SAF-1) have been reported upstream of matrix metalloproteinase 14 (MT1-MMP or MMP14) [44]. Interestingly, the MT1-MMP gene is up-regulated in colon carcinomas mediated by a direct interaction of β-catenin/TCF4 complex and their 5' flanking region, indicating that it is a direct target of Wnt pathway.

In summary, besides the presence of LEF1/TCF1 complex, some of the most relevant transcriptional regulators used by the predictor have been previously described to be associated to the regulatory regions of genes that also respond to Wnt canonical pathway, suggesting that changes in gene expression of the new Wnt/β-catenin pathway target genes can involve other factors acting on promoter regions of these genes.

### Gene Ontology analysis of new Wnt/β-catenin pathway target genes

**Figure 2:**
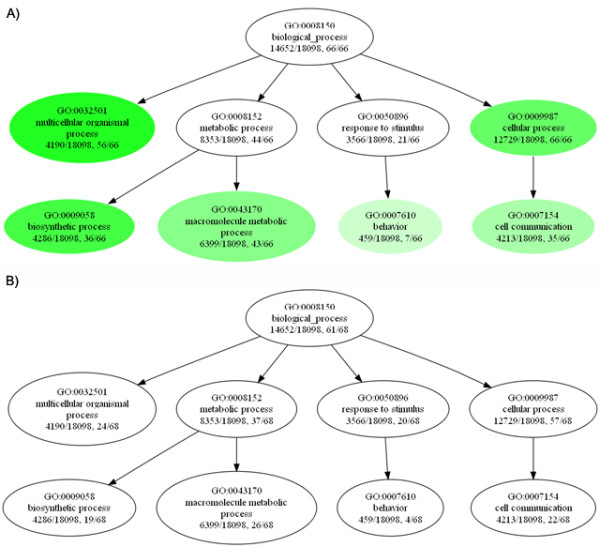
**Gene Ontology enrichment for training and proposed Wnt/β-catenin pathway targets**. Enriched nodes are colored in different intensities of green depending of adjusted p-values. Thus, more significant enrichment corresponds to the more intense green. First ratio corresponds to the proportion of GO terms in the human genome and the second ratio corresponds to the proportion of GO terms in the study group. A) GO terms enrichment for the known Wnt/β-catenin pathway target genes used as training group. B) GO terms enrichment in the same terms, for the proposed Wnt/β-catenin pathway target genes.

Classical targets of the Wnt signaling pathway have been related to different biological processes ranging from development to diseases [28], and these targets mediate Wnt function in diverse cell types and tissues. In order to know in which processes our predicted targets are involved, we used Ontologizer software [45] to assign Gene Ontology (GO) terms to the 66 known and the 89 proposed Wnt/β-catenin pathway target genes, and also some other highly ranked genes in the boundary of the threshold score. We used GO-Slim annotation [46], a subset of GO terms, to avoid the large number of GO terms and get a general comparison of the annotations. This annotation was also used to obtain a general view of which ontology terms were represented in both sets of data [47]. The number of genes in each category was calculated considering the parent-child union relationship to avoid over representation of terms in each dataset, caused by the direct acyclic graph nature of GO terms. We found that the candidates to be Wnt/β-catenin pathway target genes present a similar distribution under the three ontology categories (Additional File 3, Figure S1). Only one biological process categories (membrane fusion) was not present in the proposed Wnt/β-catenin pathway target genes (Additional File 3, Figure S1-A). On the other hand, under the molecular function ontology six categories (translational regulator, lyase, ligase, isomerase, helicase and electron transport activities) were present only in the proposed Wnt/β-catenin pathway target genes (Additional File 3, Figure S1-B). The complete list of terms using full GO annotation can be found in the supplementary material files (Additional File 4). For biological process ontology, we detected an enrichment of some categories in the training set (adj. p-value < 0.1) compared against the terms for the complete human genome dataset used in CART procedure (Figure 2). None of them was significantly enriched in the new candidates group, even when in some cases the number of terms was similar like in each case. To explore in which tissue or cell type our new proposed target genes are expressed, we used gene expression data from two different human microarray platforms, GNF1H and U95 Affymetrix chips information obtained from BioGPS (former SymAtlas) [48]. When data was clustered (Pearson coefficient distance and average linkage) to produce similar profiles, we found a group of genes in the same cluster (Additional file 5). This group represents the gene expression data from 18 cerebral tissues in one platform (GNF1H, using two normalizations approaches: MAS5 and gnRMA) and 10 cerebral tissues in the other one (U95A). Because the Wnt signaling pathway is present in several biological processes, these expression profiles led us to consider in which processes our Wnt/β-catenin pathway target genes could be represented.

To obtain more information regarding the bests ranked genes represented in these clusters, we selected a subgroup of genes activated in the two databases (Table 2). One of the new proposed targets, *tropomyosin alpha*, is involved in the contractile system of striated and smooth muscles and in non-muscle cells, and tropomyosin forms an integral part of the cytoskeleton and is involved in the regulation of cellular contraction [49]. In particular, tropomyosin is associated with neurofibrillary pathology of AD [49]. Within this cluster, we identified other interesting genes that could be involved in neuroprotection against Aβ toxicity. *Calcium/calmodulin-dependent protein kinase IV *(*CamK4*) gene is up-regulated in response to both Wnt ligands and lithium [50] to promote neuronal survival. Another candidate we identified was *synapsin 2 *(SynII). Synapsin is an abundant synaptic vesicle protein that belongs to a family of neuron-specific phosphoproteins that are highly concentrated in pre-synaptic nerve terminals. Synapsin is associated with the cytoplasmic surface of synaptic vesicles, playing a key role in neurotransmitter release and in the formation and maintenance of synaptic contacts among central neurons [51]. Synapsin II co-precipitates with fibrillar Aβ, and there is a regional loss of synapsin I in the hippocampus of patients with late-stage AD [52,53], suggesting that the regional decrease in synapsin is associated with cytoskeletal changes as well as with Aβ deposits [54]. Interestingly, *Ryk *(receptor related to tyrosine kinase) gene was also found as a putative Wnt target gene. Ryk functions as a co-receptor with Frizzled for Wnt ligands through the activation of a β-catenin-independent signaling pathway [55-58]. In fact, Ryk is able to bind to Dishevelled, thereby activating the canonical Wnt pathway. Ryk function is related to axon guidance and neurite outgrowth, making it an interesting target for Wnt activation [59,60].

**Table 2:** A sample of predicted Wnt/β-catenin pathway target genes

Entrez ID	Symbol	Name	Score
814	CAMK4	*calcium/calmodulin-dependent protein kinase IV*	1489
84152	PPP1R1B	*protein phosphatase 1, regulatory (inhibitor) subunit 1B (DARPP-32)*	489
8503	PIK3R3	*phosphoinositide-3-kinase, regulatory subunit 3 (p55, gamma)*	310
27124	PIB5PA	*phosphatidylinositol (4,5) bisphosphate 5-phosphatase, A*	196
7168	TPM1	*tropomyosin 1 (alpha)*	422
8871	SYNJ2	*synaptojanin 2*	316
1917	EEF1A2	*eukaryotic translation elongation factor 1 alpha 2*	384
3705	ITPK1	*inositol 1,3,4-triphosphate 5/6 kinase*	213
6854	SYN2	*synapsin II*	184
10236	HNRPR	*heterogeneous nuclear ribonucleoprotein R*	258
8507	ENC1	*ectodermal-neural cortex (with BTB-like domain)*	177

Among the new target genes, we also found a member of the phosphatidylinositol phosphatases, *synaptojanin 2 *(*Synj2*). A spliced form of the mRNA from this gene partially overlaps the function of *synaptojanin 1 *(*Synj1*) in nerve terminals, with additional roles in neurons and other cells [61]. From the AD perspective, haploinsufficiency of *Synj1 *exerts its protective effect on oligomers Aβ-mediated down-regulation of *phosphatidylinositol (4,5) diphosphate phosphatase 2 *and impairment of synaptic function [62]. In fact, other genes involved in inositol metabolism, *phosphatidylinositol (4,5) bisphosphate 5-phosphatase A *(*PIB5PA*), or in phosphatidylinositol signaling, as *inositol 1,3,4-triphosphate 5/6 kinase *(*ITPK1*) and *phosphoinositide-3-kinase regulatory subunit 3γ *(*PIK3R3*), were identified by our method, which is consistent with the potential connection between AD and phosphoinositides [63].

Another interesting Wnt-regulated gene candidate is the *inhibitory subunit for protein phosphatase 1 *(*PPP1R1B*), a dopamine- and cAMP-regulated phosphoprotein also known as *DARPP-32 *[64]. Through its inhibition of *protein phosphatase 1*, *DARPP-32 *controls the state of phosphorylation and the activities of several key proteins, including ion channels, ion pumps, neurotransmitter receptors, and transcription factors; thus, *DARPP-32 *controls the physiological characteristics of neurons containing dopamine receptors [65]. Interestingly, inhibition of *protein phosphatase 1 *strongly stimulates soluble amyloid precursor protein (sAPP) secretion and inhibits Aβ formation [66]; therefore, Wnt-dependent up-regulation of *DARPP-32 *provides a potential route for the prevention of AD.

Another new target is *heterogeneous nuclear ribonucleoprotein R*. Products of these families of genes play important roles in regulating neural-specific pre-mRNA splicing, thereby contributing to the regulation of neural function and development [67]. Therefore, their neuron-specific regulation and function reveals new insights into physiological and pathological events. *Ectodermal-neural cortex 1 *(*ENC-1*), which is a component of the TCF/β-catenin complex, is another new target that is up-regulated in colorectal carcinomas [68]. Interestingly, ENC-1 is a nuclear matrix protein abundantly expressed in the brain and appears to be localized in primary neurons and is up-regulated in brain tumors, suggesting that it might be involved in brain tumorigenesis [69]. These results suggest that the Wnt pathway can transcriptionally modulate a neuroprotective response against Aβ peptide, promoting neuronal survival and rescuing changes in activation or sub-cellular localization of Wnt components [21].

### Biological validation

To test whether new targets of the Wnt/β-catenin pathway predicted by our method change their expression depending of pathway activation we used RT-qPCR [70]. We measured changes in mRNA levels between HEK-293 that over-express and secrete Wnt ligands and HEK-293 wild-type cells. Table 3 shows expression fold-change for seven genes assigned to class 1 by the method and covering a wide range of scores, and 4 genes not selected as Wnt/β-catenin pathway targets by the method. Two of them appear in the first 2% of the highest scores and two are controls with very low scores. We observe that five of the nine genes analyzed, having different scores in our method, exhibited significant changes in gene expression. Among them, a link between AD and phosphoinositides has been previously mentioned for *IPTK1 *and *PIK3R3*. Also, we found significant differences in the expression of *adrenomedullin *(*ADM*), a peptide involved in a wide range of physiological and pathological processes [71]. In particular, ADM prevents damage caused by oxidative stress through the phosphatidylinositol-3 kinase-dependent pathway, and it was recently [72] reported that lack of ADM in mouse brain results in behavioral changes. On the other hand, high levels of ADM in plasma have been found in patients with chronic Schizophrenia [73], which confirms its role in brain disorders. Two other new target genes that also change their expression levels are involved in neuronal and brain development. The first one, *CRKL*, is part of the Reelin pathway, which is necessary for embryonic development of the cerebral cortex, cerebellum and hippocampus [74]. In particular, *CRKL *is required for dendritogenesis but not for axonogenesis in cultured hippocampal neurons cells [75]. Mice lacking expression of a *CRKL *ortholog exhibits several defects in cardiac and neural crest formation during early stages of development. *CRKL *exerts its function as a scaffold protein interacting with phosphorylated tyrosine domains of *dab-1 *effector protein. The last gene is *Neurogenin 3 *(*NEUROG3*) that is involved in gliogenesis during the development of the central nervous system [76]. In particular, over-expression of *NEUROG3 *changes the morphology of dendrites in hippocampal neurons through reduction of synaptic contacts [77].

**Table 3:** Gene expression change measured by RT-qPCR for 9 predicted Wnt/β -catenin targets and 2 controls

Entrez ID	Symbol	Name	CART Score	Fold Change
133	ADM	*Adrenomedullin*	260	1.91*
1399	CRKL	*v-crk sarcoma virus CT10 oncogene homolog (avian)-like*	138	2.57*
1917	EEF1A2	*eukaryotic translation elongation factor 1 alpha 2*	384	1.13¥
3705	ITPK1	*inositol 1,3,4-triphosphate 5/6 kinase*	213	1.84*
8503	PIK3R3	*phosphoinositide-3-kinase, regulatory subunit 3 (p55, gamma)*	310	1.47*
8507	ENC1	*ectodermal-neural cortex (with BTB-like domain)*	177	1.31¥
27242	TNFRSF21	*tumor necrosis factor receptor superfamily, member 21*	404	1.14¥
50674	NEUROG3	*neurogenin 3*	586	1.90*
11040	PIM2	*pim-2 oncogene*	266	1.18
334	APLP2	*amyloid beta (A4) precursor-like protein 2*	Ctrol	1.06
9997	SCO2	*SCO cytochrome oxidase deficient homolog 2 (yeast)*	Ctrol	1.03

*p-value < 0.05, ¥ *involved in brain development or abnormalities (see text for references)*.

Results from RT-qPCR revealed that, at different scores (even after the first 1%), the assignment of CART procedure identified several new targets for the Wnt/β-catenin pathway that exhibit changes in their expression levels in response to Wnt ligand.

## Discussion

The majority of known genes regulated through the Wnt and TCF/β-catenin pathways are important in developmental and differentiation mechanisms in complex organisms. The training set selected for the proposed multiple CART strategy is based on this information, and Gene Ontology analysis revealed that this group of genes is enriched in categories like multicellular organismal processes, which involve GO terms like cell differentiation, embryonic development and anatomical structural development with respect to the whole-genome ontology information. The genes selected by our method have a similar distribution of ontology terms but no enrichment was found. The fact that both groups of genes can be distributed in the same ontologies, lead us to argue that the loss of enrichment is more related with the change in the number of genes analyzed than the genes in the group of candidates themselves. Indeed, the Benjamini-Hochberg method for multiple testing corrections uses the length of the data to adjust the p-value. Clustering of gene expression results revealed that our predicted new Wnt/β-catenin pathway target genes in brain tissues have expression profiles similar to those of colorectal cancer cell lines, which have high levels of accumulated β-catenin. This similarity suggests a conserved mechanism to regulate the expression of these genes, based in β-catenin and TCF/LEF interactions. Our RT-qPCR results indicated that some of the new Wnt/β-catenin pathway targets assigned using the multiple CART method change their expression levels when Wnt/β-catenin ligands are over-expressed. The physiological role for some of these new targets revealed that these genes are particularly involved in brain pathologies. We also associated the database from Hatzis et al. [32] (containing TCF4 chromatin occupancy data) with our predicted new candidates involved in the Wnt/β-catenin pathway and we found that 24 genes from our list had been detected in a ChIP-coupled DNA microarray. *TNFRS21 *is an example of a gene that did not change its expression level in the presence of exogenous Wnt expression; however, this gene has been reported as Wnt target by ChIP with colorectal cancer cell lines and TCF4 antibodies [32]. Remarkably, *TNFRSF21 *receptor gene, also known as *Death receptor 6 *(*DR6*), is widely expressed in neurons and regulates axon pruning and neuronal death [78]. In addition to the role *DR6 *plays in neuronal development, APP was also identified as a ligand for this receptor, and their interaction activates a caspase-dependent self-destruction program, suggesting that extracellular fragments of APP may contribute to AD [78].

Other genes assigned as Wnt/β-catenin pathway targets by the proposed method may be involved in brain or nervous systems abnormalities other than AD. For example, *elongation factor 1 alpha 2 *(*EEF1A2*) corresponds to an isoform belonging to the elongation factor family complex. This isoform has been predominantly detected in brain, heart and muscle tissues [79] and has been implicated as an oncogene in ovarian and breast tumors [80,81]. A spontaneous autosomal recessive mutation in *EEF1A2 *is responsible for the *waste *phenotype in mouse, which, among others effects, shows abnormalities in the spinal cord and brain stem and leads to severe motor neuron degeneration [82,83]. Finally, the oncogene *PIM2*, which is dysregulated in acute leukemia [84], has recently been shown to be up-regulated in a microarray screen using post-mortem brain-derived microglia [85]. The expression level of *EEF1A2 *and *PIM2 *did not change in our RT-qPCR measurements, suggesting that another condition or synergistic signaling pathway is required to regulate their expression in response to Wnt ligands. In this direction, the fact that some of the relevant transcription factors used by the proposed method have been described acting together with β-catenin supports the idea that other regulators besides LEF1 can control gene expression. More interesting, the presence of nuclear receptors as GR and HNF4α suggests a crosstalk between hormone regulation and Wnt/β-catenin pathway to control gene expression in brain.

A more detailed analysis of each one of these Wnt/β-catenin target genes is required to prove in vivo whether their change in gene expression levels is a direct response to Wnt pathway activation or corresponds to more complex signaling networks that operate in brain tissues. However, to our knowledge, this study represents the first approach to use *cis *regulatory module information and a CART strategy to propose a global analysis to detect new genes involved in brain diseases and AD.

## Conclusions

In this study, we developed a method to identify new Wnt/β-catenin pathway target genes based on the analysis of the number of times that each of 432 selected transcription factors binding site motifs appear in the promoter region of the genes (1,000 bp upstream and 200 bp downstream of the gene transcription start site) in the human genome. We developed a strategy based on the use of the CART method to detect genes in which the presence of TF binding site motifs is similar to the one in the known targets of the Wnt/β-catenin pathway.

Through the classification method, we proposed 89 new candidates in which transcriptional regulation could be controlled under the canonical Wnt/β-catenin pathway. The tissue-specific expression profiles of these genes revealed a common pattern between brain tissues and, notably, are similar to colorectal carcinoma profiles, where increased levels of cytoplasmic β-catenin are responsible for activating expression of several groups of genes [31]. In agreement with these results, several of the new candidates bind to LEF1, and it is well established that TCF/LEF1 forms a complex with β-catenin to activate the expression of genes under the canonical Wnt pathway [28].

The Wnt pathway potentially promotes neuronal survival and rescues deficiencies resulting from alterations in the expression of Wnt components [21]. Among the new candidates, we found several genes that could be regulated through the canonical Wnt/β-catenin pathway, and their function is to regulate neuronal physiology and brain tumorigenesis, to decrease the levels of Aβ fibrils and to promote soluble APP forms. Further analysis is required to demonstrate a direct interaction between TCF/LEF1 transcription factors with the promoter regions of these genes in response to the activated Wnt/β-catenin, but the predictions made by the proposed CART method revealed a strong connection between the canonical Wnt pathway and the cooperative control of gene transcription in brain physiology and neuropathology, in particular AD.

## Methods

### Multiple CART predictor algorithm

In this work we developed a supervised classification method; that is, a method that uses a set of examples with known classification to train a predictor, which can then be used to classify a new sample. In this case, we classified human genes using as characteristic variables the number of times each TF binding site motif from a list of 432 motifs obtained from [86] occurs in the upstream region of the gene (more precisely, 1,000 bp upstream and 200 bp downstream of the gene transcription start site, but for simplicity we call it the "upstream region"). We classified with respect to two classes: class 1 containing known Wnt/β-catenin pathway target genes and class 2 containing the remaining genes.

The group of Ron Shamir [86] used PRIMA and position weight matrices for 432 TRANSFACT motifs to perform a footprinting analysis of 15,476 human promoters for the region between 1,000 bp before and 200 bp after the transcription start site. These motifs are associated to 290 transcription factors. We downloaded their data from http://acgt.cs.tau.ac.il/prima/PRIMA.htm and built a matrix of 15,476 rows (representing genes) by 432 columns (representing TF binding site motifs), where each entry in the matrix contains the number of times each motif was found in the upstream region of the gene (Additional File 6).

We obtained from The Wnt Homepage [38] a list of 107 known target genes for the Wnt pathway and we filtered it to select β-catenin/LEF1 target genes. To further refine this list, we selected only human genes in which LEF1 interaction with the promoter regions has been experimentally proven using electrophoretic mobility shift assays, ChIP or reporter assays. Finally, 66 genes (Additional File 1) were considered as the initial set of Wnt/β-catenin pathway target genes. We observe that these genes were included in the 15,476 genes obtained from Shamir's web page.

**Figure 3:**
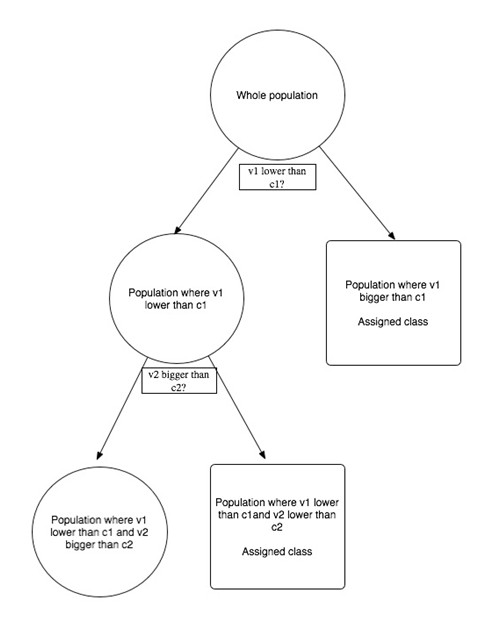
**Classic structure of a CART tree**. The first node of the tree is subdivided into two finer nodes depending on whether v1 is lower than c1. The resulting nodes are further subdivided to determine the assigned class.

Our classification method proposes the use of a series of 1,500 "Classification and Regression Trees" (abbreviated CART). This last is a supervised classification method which builds, based on a training sample, a hierarchy of variables that are used to recursively split the sample into coherent groups, until a certain degree of separation of the sample is achieved. This chain of splits can be visualized as a binary tree (called CART tree), which can be used to classify a new individual. In our case, each internal node of the tree (including the root and excluding the leaves) is associated to a TF binding site motif identifier and a threshold value c (Figure 3). The leaf nodes are only associated to a class label. The classification of an individual gene starts at the root node. If the TF binding site motif corresponding to the node appears more than c times in the upstream region of the gene, one moves to the node corresponding to the "right child", if not, one moves to the "left child". This procedure continues until a leaf node is reached, and its class label defines the class attributed to the gene.

To start the training of a single CART tree, we put in class 1 the 66 genes experimentally validated as Wnt/β-catenin pathway targets described above, and we formed class 2 with 8,000 genes randomly generated among the remaining 15,410 genes. These 8,066 genes are associated to the root node of the tree. The training algorithm, described in detail in [87], partitions at each iteration the training set associated to an internal node into two groups according to a particular choice of a TF binding site motif and a threshold c; one determines a subset of genes associated to the left child and the other one a subset of genes associated to the right child as explained before. The splitting (i.e. selection of the TF binding site motif and the threshold c associated to the node) is performed in such a way that the "average Gini impurity" < G > is minimized, where:

N_L_, N_R _are the number of genes associated to the node we are splitting that are candidates to pass to the left and to the right branches respectively, and for b = L and b = R

with N_b,1 _and N_b,2 _the number of genes initially in class 1 and class 2 moving to branch b respectively. The change in the Gini impurity can be interpreted as a measure of the effectiveness of a given TF binding site motif to characterize or determine the class of a gene. That is, in our context, if it is a target of the Wnt/β-catenin pathway. This algorithm stops once the Gini impurity decrease is zero at all leaf nodes. We observe that no pruning is achieved at this step. In fact, to obtain robust predictions for the whole population we used "sample test": only a part of the labeled sample is used to construct the first tree and the rest is used to test and to prune the tree, searching a minimal misclassification. Thus, after this first tree was constructed, we started the evaluation and pruning of the tree using a second set of 8,000 randomly chosen genes. We put in class 2 the set difference of this new collection with the first randomly chosen set of 8,000 genes (approx. 4,000 genes remain in practice), and in class 1 remained the same previously used 66 genes since its size is too small. Essentially as in the classical theory of CART [87], in this step we used the option of considering only splits whose impurity decrease is greater than a given cost to obtain a sub-tree (or pruned tree) of the first one. We kept the sub-tree that minimizes the classification error in this second set of genes. This finishes the procedure to build a single CART tree.

Using the procedure described above we built 1,500 independent CART trees and each one of the 15,476 genes was classified either in class 1 or class 2 by each tree. The *score *of a gene was defined as the number of trees among these 1,500 that classify it in class 1. Another criterion is to use Random Forest [88].

**Figure 4:**
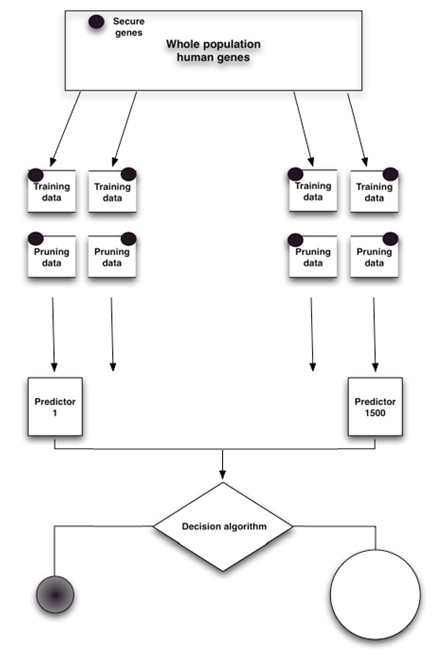
**General structure of the proposed method**. We trained 1,500 CART trees using 66 known target genes marked as black dots and two groups of 8,000 randomly chosen genes from a list of 15,476 genes in the human genome. The first group is used to produce a first tree and the second to prune and evaluate it. The classification method is the consolidation of the results of the 1,500 CART trees.

Finally, we considered a gene as a candidate to be a Wnt/β-catenin pathway target if its score was above a threshold C (Figure 1). We chose a simple way to determine the value of C. That is, we ordered genes with respect to their scores and C is the value determining the highest percentile. A general view of the method is provided in Figure 4. This method was implemented on the R statistical package using rpart library. To accelerate the procedure the implementation considers the use of a distributed system of computers. It is available in Additional File 7.

### Ranking of transcription factors

To quantify the importance for the classification of each TF binding site motif we analyzed each one of the 1,500 CART trees produced by the method. We considered the number of times a TF binding site motif was associated to a tree and the depth of the nodes it is associated. Then we defined two indexes. The first one (I_1_) was used to rank "primary variables", i.e. more weight is given to the variables used to partition the population near the root. For each TF binding site motif *m*, it is given by the following formula:

where *NT_i_(m) *is the set of internal nodes of the ith-tree associated to *m *and *depth(n) *is the depth of node *n *in such tree. The second index (*score*) simply counts the number of trees where each motif was used.

Then we constructed two rankings, one for each index. With both we found interesting biological interpretations. In Table 1 we show the TFs that appeared to be more relevant from the biological point of view using both criteria.

### Cross-validation of the method

In the discussion below we already analyzed the biological relevance of the results of the application of our strategy. From the methodological point of view, we used the "leave-one-out cross-validation" methodology to study how sensitive is the proposed method to detect known Wnt/β-catenin pathway target genes. The leave-one-out cross-validation was applied as follows: one of the 66 known Wnt/β-catenin pathway target genes was isolated and the remaining 65 genes were used to train the multiple CART predictors as described before. After, we generated the list of proposed new Wnt/β-catenin pathway target genes using the highest percentile criterion and we computed the indexes of the variables. We obtained that 100% of the known Wnt/β-catenin pathway target genes were correctly classified when not considered in the training set, and at least 144 (93%) of the predicted target genes were the same as when no gene was excluded from the training set.

We also evaluated the robustness in relation to changes in the training sets by performing four independent instances of our method and comparing their predictions. Over 95% of the proposed genes are recovered when our method is used with different samplings, and the known Wnt/β-catenin genes are always detected as seen in Table 4. Among the coincidences the most relevant were *calcium/calmodulin-dependent protein kinase IV *(*CamK4*), for which there exist strong evidences for up-regulation in response to both Wnt ligands and lithium [50], and *tropomyosin 1 (alpha) *that is associated with neurofibrillary pathology of AD [50].

**Table 4:** Comparative analysis of the method and robustness

Method	Instance 1	Instance 2	Instance 3	Instance 4	Prior	New
Instance 1	155 (100%)	150 (97%)	147 (95%)	151 (97%)	66 (100%)	89
Instance 2	150 (97%)	155 (100%)	147 (95%)	150 (97%)	66 (100%)	89
Instance 3	147 (95%)	147 (95%)	155 (100%)	149 (96%)	66 (100%)	89
Instance 4	151 (97%)	150 (97%)	149 (96%)	155 (100%)	66 (100%)	89
KNN 1	1 (1%)	1 (1%)	1 (1%)	1 (1%)	0 (0%)	30
KNN 2	0 (0%)	0 (0%)	0 (0%)	0 (0%)	0 (0%)	17
SVM	66 (43%)	66 (43%)	66 (43%)	66 (43%)	66 (100%)	0
CART	58 (37%)	58 (37%)	58 (37%)	58 (37%)	44 (67%)	46
L-1-O (avg)	147,8 (95%)	145,6 (94%)	144 (93%)	145,7 (94%)	66 (100%)	89

### Comparison with other classification methods

To compare the performance of our strategy with other classification methods, using the same data we produced classifiers with classical implementations of K nearest neighbours method (KNN), for K taking values from 1 to 5, Support Vector Machine (SVM) method, with radial basis, and standard CART method, as implemented in the R statistical platform in the libraries 'class', 'e1071' and 'rpart'. All data (15,476 genes) was classified using those methods (see results in Additional File 1) and we computed the sensitivity of classifying known Wnt/β-catenin pathway target genes, as shown in column 'Prior' in Table 4. KNN was performed using directly 'knn.cv' routine (which also implements a leave-one-out test) over all data and it did not recover the known Wnt/β-catenin pathway target genes. When K = 1 this method proposed 30 candidates and only one of them coincides with our predictions; when K = 2 there are 17 proposed target genes, none of them coincides with our prediction; and for K greater than or equal to 3 all genes were classified as non-targets. The SVM method (trained using 10-fold cross-validation) recovered all known target genes but all others were classified in class 2 of non-target genes. This is probably a result derived from over-fitting, which is expected given the huge asymmetry between the two classes. The single CART was trained using all 66 known Wnt/β-catenin target genes and a sample of 8,000 genes not a priori related to this pathway, and then pruned as described previously. In this case 44 of the 66 known Wnt/β-catenin pathway target genes were recovered and 46 new targets were proposed. The coincidence with our method was 37%, that is, 58 genes appeared in all instances of our method. Table 4 summarizes the coincidences of these methods and indicates the number of known genes recovered by each one.

### Database analysis of tissue-specific gene expression and Gene Ontology analysis

Gene Ontology analysis was performed using Ontologizer software [46]. Overrepresentation of GO terms was calculated considering the parent-child intersection relationship [89]. Benjamini - Hochberg FDR procedure for multiple test correction was applied.

The Wnt target candidate tissue expression was analyzed using the online tool BioGPS available at http://biogps.gnf.org[90]. This database represents an extensive collection of data from human gene expression samples across a diverse array of tissues, organs and cell lines. These samples were predominantly obtained from physiologically normal humans, and thus this dataset represents a preliminary but substantial portion of the normal mammalian transcriptome [91]. Gene expression data values were downloaded from two microarrays platforms: GNF1H with MAS5 and gcRMA normalization procedures and U95A. Data were used to perform cluster analysis considering Pearson distance and average linkage.

### Biological validation

We chose HEK 293 as our experimental model as it has been widely used as an appropriate cellular model for Wnt activation experiments. In this study we have used a stable clone for the expression of Wnt-3a ligand, and for this purpose we have considered HEK 293 cells that have been successfully used in the past for Wnt ligand production [92-94]. Interestingly, initial results obtained by DNA microarray analysis of HEK 293 cells showed that 293 cells stain strongly and specifically with antibodies to several neurofilament proteins, which are generally thought of as excellent markers for neuronal lineage cells [95]. Two cell lines were generated to verify the response of predicted Wnt targets. Human embryonic kidney 293 (HEK-293) cells were stably transfected with a pcDNA/Wnt expression vector to ensure over-expression of Wnt ligands. The same cells were also transfected with empty vector pcDNA as a control. Anti-Hemagglutinin epitope tag (HA) immunodetection was used to confirm the presence of Wnt ligands in the media as previously described in [96]. RNA from both cultures was extracted using the TRI Reagent kit (Ambion) according to the manufacturer's instructions. RNAs were then treated with an RNase-free DNase set (Qiagen) and reverse transcribed with Oligo-dT and Superscript II (Invitrogen). RNA isolated from HEK-293 cells was spiked (1:2,000) with RNA synthesized from *Bacillus subtilis *Dap (ATCC 87486) as a control to normalize replicates. Changes in mRNA levels were determined by RT-qPCR using LightCycler real-time PCR system (Roche). The primers are shown in Table 5. Real-time amplification data were analyzed using the DD-CT method [97], and statistical significance was determined by a t-test.

**Table 5:** List of primers used for RT-qPCR in this study

Entrez ID	Symbol	Primer Sense (5'- > 3')	Primer Antisense (5'- > 3')
133	ADM	TGGGTTCGCTCGCCTTCCTA	CATCCGCAGTTCCCTCTTCC
1399	CRKL	TGATTCCTGTCCCTTATGT	GGTCTGAGGTTGAGCGTAT
1917	EEF1A2	CCTTCAAGTATGCCTGGGTG	CAGTCCGCCTGGGATGTAC
3705	ITPK1	CGGCTTGACTTTCCCATTC	CTCGCCAACCACGAACACC
8503	PIK3R3	CATTACCAGCAGACATCC	CTCTTCCCACTTCCTCTTT
8507	ENC1	TGGGAGATGTGACAGCAA	CAGTAGGAATCAGCGAGTA
27242	TNFRSF21	CCCACAGGACAAGAACAA	AGCCGCTGGATGTAGAGT
79962	DNAJC22	CAGCTTGAGGGTCTAAGGATA	GGTTACTCGCAGCACAGAA
50674	NEUROG3	GGCTGTGGGTGCTAAGGGTAA	CAGGGAGAAGCAGAAGGAACAAG
11040	PIM2	CTCAGCCCAGGATTCTTTA	AGAGCACTTGGGATAACAGA
334	APLP2	GTGGAATAGGGAACTGTAAT	GGGGAAGTGAACGGTAAAA
9997	SCO2	AGTGGGTGCTGATGTACTTTG	CGCAGCCCGTTTAATGATGG

## Authors' contributions

CH selected the training group for the CART method, gene ontology analysis, new targets analysis and drafted the manuscript. RA implemented the CART method and drafted the manuscript. LP contributed to primer design and RT-qPCR analysis. MC contributed to Wnt over-expression cell culture and initial data analysis. AA contributed to design, implementation and data analysis of CART procedure and corrected the draft. MG, SM, NI and AM participated in the design and coordination of the study. All authors have read and approved the final manuscript.

## Supplementary Material

Additional file 1 **New proposed Wnt/β-catenin canonical pathway target genes selected by multiple CART method**. The file contains the list of 15,476 genes classified by the different methods discussed in the text. In particular the 89 new Wnt/β-catenin targets predicted by our method and the 66 known genes used in the training procedure. Click here for file

Additional file 2 **Ranking of the variables used by the proposed method**. The file contains for each one of the 432 TF binding site motif its ranking according to: frequency of use and index I_1 _as described in the text. Click here for file

Additional file 3 **Gene ontology analysis of known and proposed Wnt/β-catenin pathway target genes**. The file contains the GO analysis count performed on known and predicted Wnt/β-catenin targets. Click here for file

Additional file 4 **Gene ontology terms for proposed Wnt/β-catenin pathway target genes**. The file contains the complete GO terms for the predicted Wnt/β-catenin pathway target genes. Click here for file

Additional file 5 **Cluster of gene expression for proposed Wnt/β-catenin pathway target genes**. The file contains the heat map results from cluster analysis using data from BioGPS database. Click here for file

Additional file 6 **Human footprinting matrix used in the CART procedure**. The file contains the original data file obtained from [86], the code used to transform them to files "genes.txt", "motifs.txt" and "upstream.txt", and the code used to load them to R data format. File "upstream.txt" contains a text plain matrix with genes in rows and predicted transcription factor binding site motifs in columns, adopting the value 0 when no TF binding site motif is predicted or the number of TF binding site motifs predicted by PRIMA. Click here for file

Additional file 7 **Function codes and routines implemented in R for the CART procedure**. The file contains the files used in CART procedure: onetree.R is used to train a single tree, consolide.R is used to summarize the 1,500 trees to obtain the gene score and rankvars.R is used to determine the score of the variables (TF binding sites motifs). Finally, Makefile is used to coordinate all computations using GNU make. Click here for file
