# Supplementary material for: Genome-wide identification of new Wnt/β-catenin target genes in the human genome using CART method
Source: BMC Genomics. 2010 Jun 1;11:348. doi: 10.1186/1471-2164-11-348 (PMC2996972; doi:10.1186/1471-2164-11-348)
Supplement: Additional file 5 — Cluster of gene expression for proposed Wnt/β-catenin pathway target genes. The file contains the heat map results from cluster analysis using data from BioGPS database. [file 1471-2164-11-348-S5.PPT]

## Slide 1
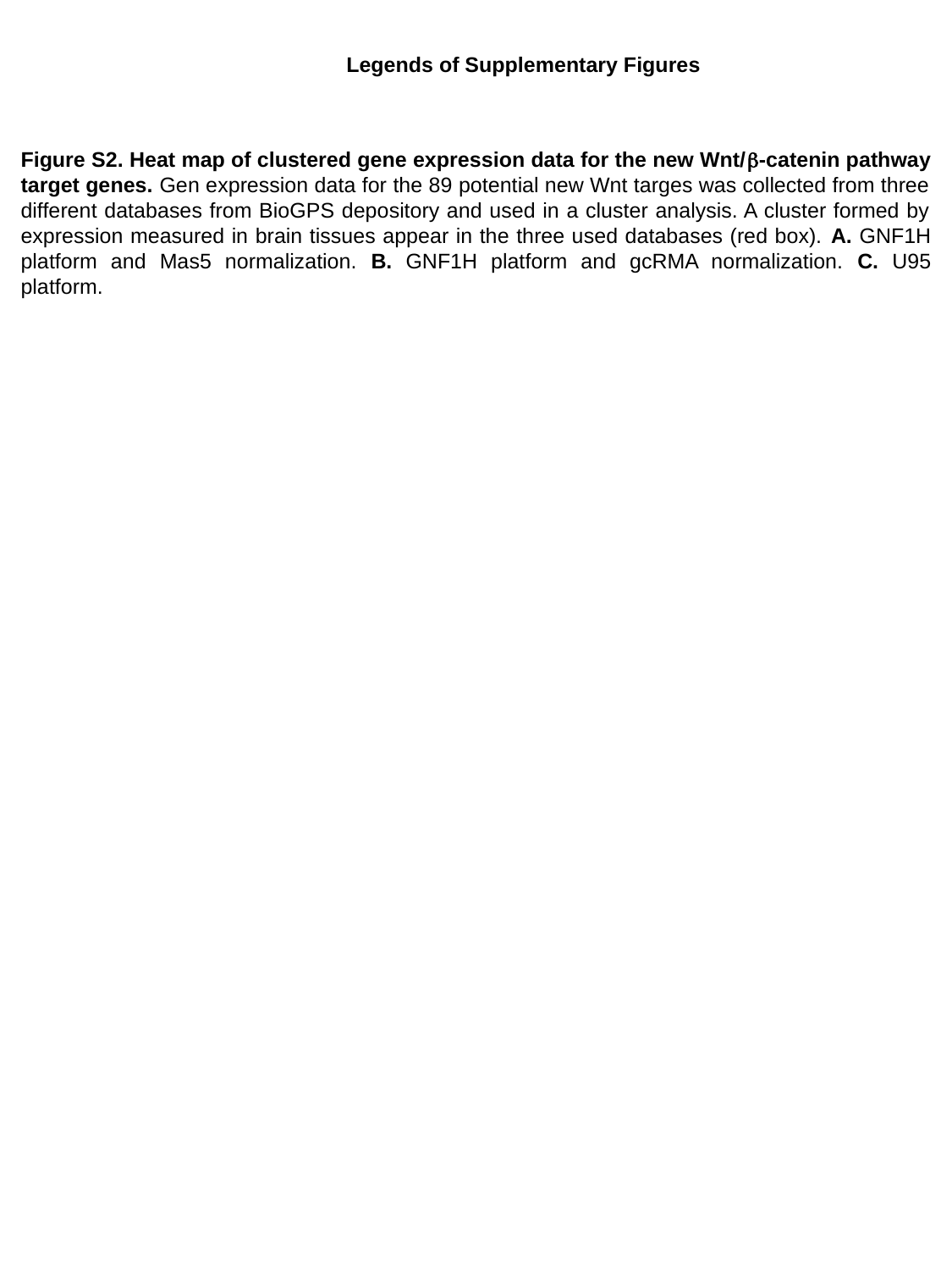

Legends of Supplementary Figures
Figure S2. Heat map of clustered gene expression data for the new Wnt/β-catenin pathway target genes. Gen expression data for the 89 potential new Wnt targes was collected from three different databases from BioGPS depository and used in a cluster analysis. A cluster formed by expression measured in brain tissues appear in the three used databases (red box). A. GNF1H platform and Mas5 normalization. B. GNF1H platform and gcRMA normalization. C. U95 platform.

## Slide 2
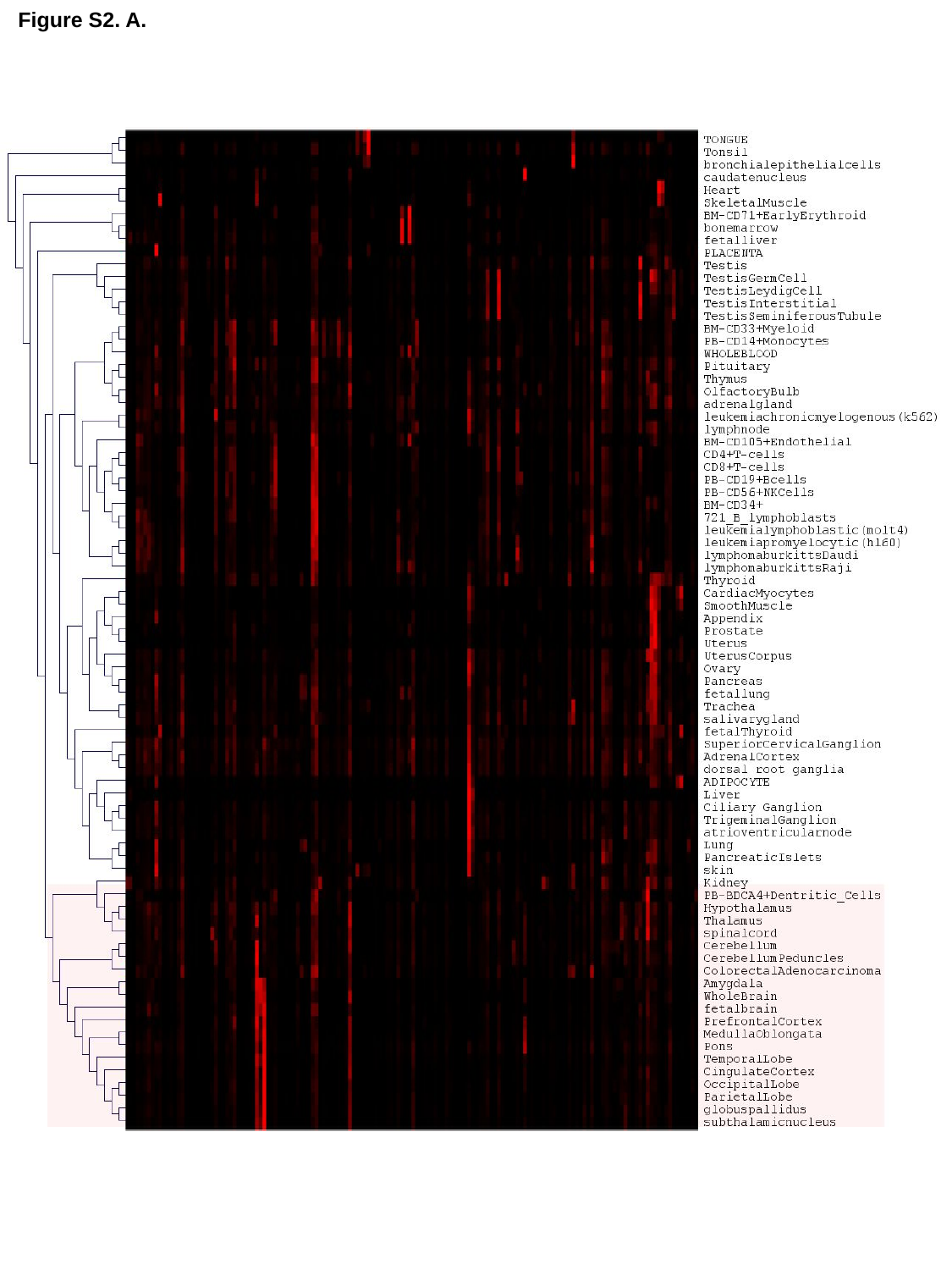

Figure S2. A.

## Slide 3
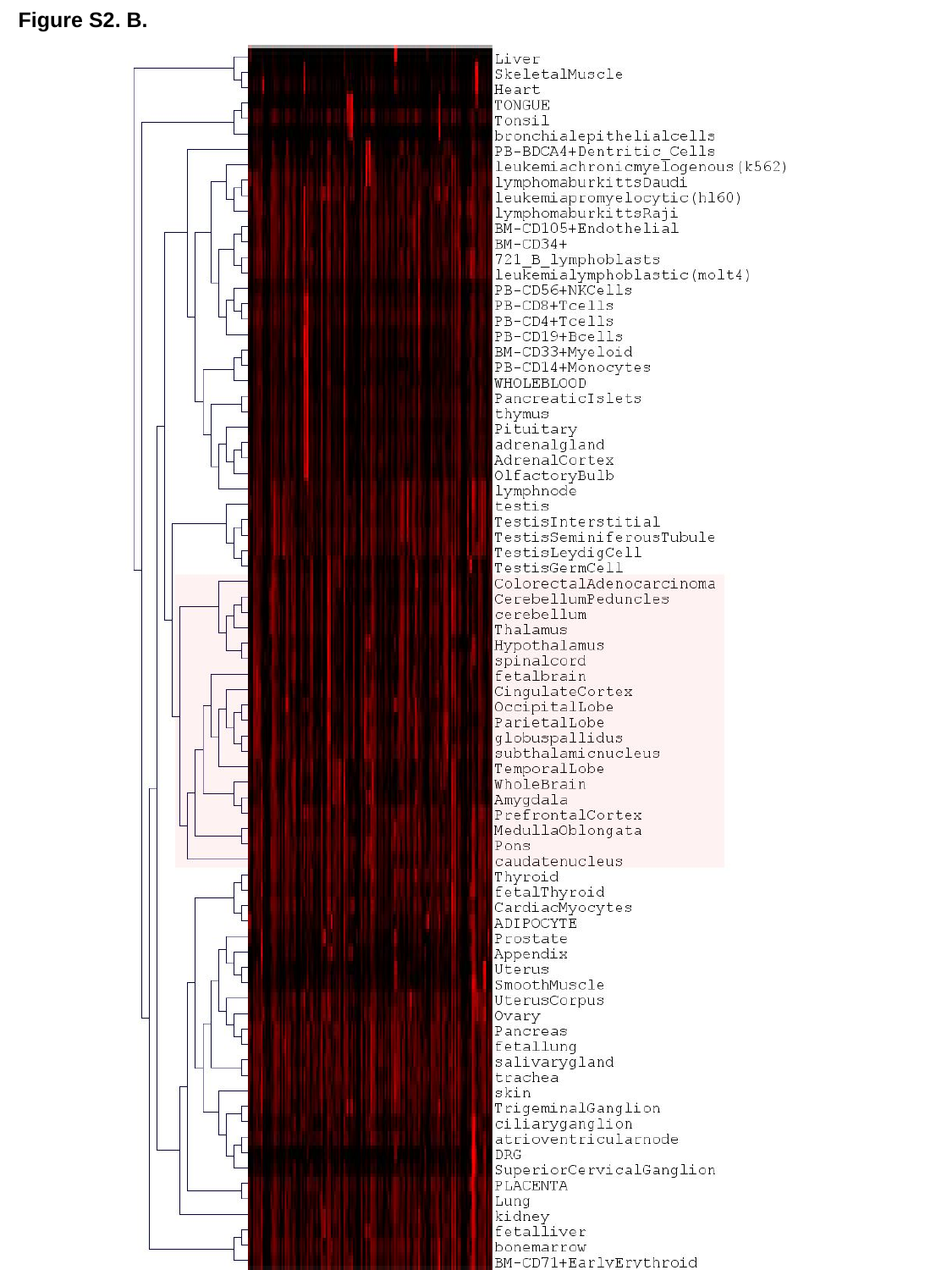

Figure S2. B.

## Slide 4
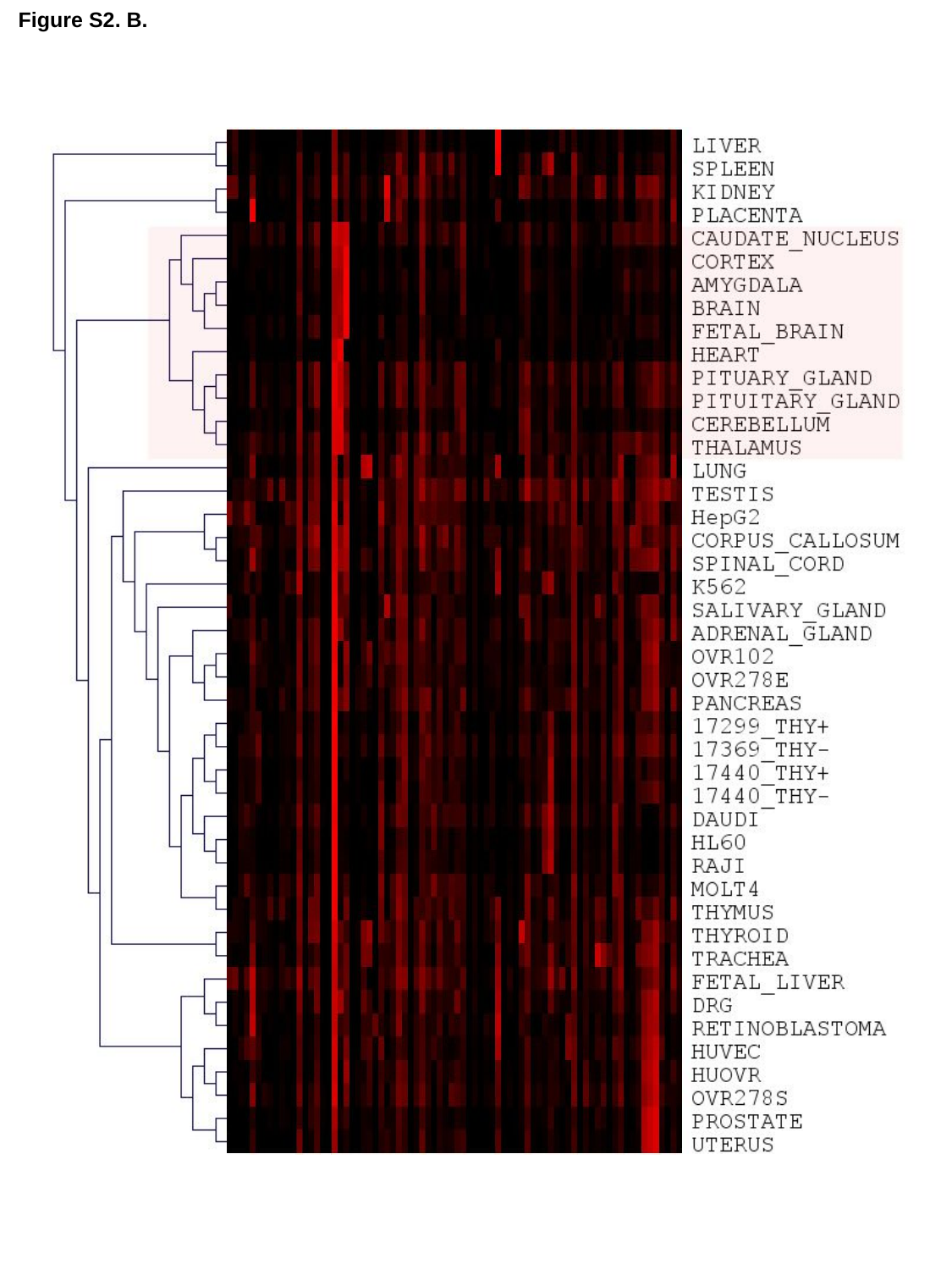

Figure S2. B.
